# {*N*,*N*-Dimethyl-*N*′-[phen­yl(2-pyrid­yl)methyl­ene]ethane-1,2-diamine-κ^3^ *N*,*N*′,*N*′′}dithio­cyanato-κ*N*,κ*S*-copper(II)

**DOI:** 10.1107/S1600536809048594

**Published:** 2009-11-21

**Authors:** Xiu-Qing Zhang, Chun-Ying Li, He-Dong Bian, Qing Yu, Hong Liang

**Affiliations:** a College of Chemistry and Chemical Engineering, Guangxi Normal University, Guilin, Guangxi 541004, People’s Republic of China

## Abstract

In the title complex, [Cu(NCS)_2_(C_16_H_19_N_3_)], the Cu^II^ atom is coordinated by a total of four N atoms; three from one tridentate Schiff base ligand and one from one of the NCS^−^ ions. The S atom from the other NCS^−^ ion completes the distorted square-pyramidal coordination.

## Related literature

For general background to Schiff base complexes, see: Shi *et al.* (2004 ▶); Chandra & Sangeetika (2004 ▶); Ramesh & Maheswaran (2003 ▶); Guo *et al.* (2009 ▶). For a description of the geometry of five-coordinated metal complexes, see: Addison *et al.* (1984 ▶).
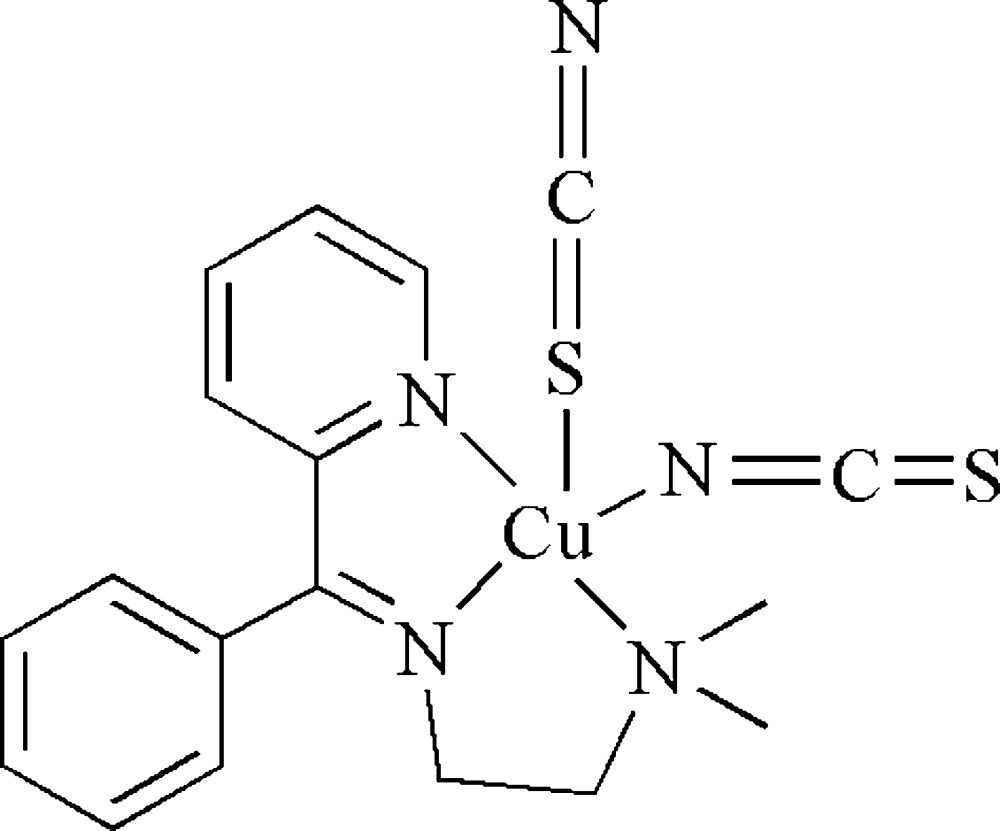


## Experimental

### 

#### Crystal data

[Cu(NCS)_2_(C_16_H_19_N_3_)]
*M* *_r_* = 433.04
Orthorhombic, 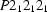

*a* = 7.6524 (13) Å
*b* = 9.2048 (15) Å
*c* = 27.931 (5) Å
*V* = 1967.4 (6) Å^3^
*Z* = 4
Mo *K*α radiation
μ = 1.33 mm^−1^
*T* = 294 K
0.30 × 0.24 × 0.20 mm

#### Data collection

Bruker APEXII CCD area-detector diffractometer
Absorption correction: multi-scan (*SADABS*; Sheldrick, 1996 ▶) *T*_min_ = 0.748, *T*_max_ = 1.000
11093 measured reflections
3978 independent reflections
3035 reflections with *I* > 2σ(*I*)
*R*_int_ = 0.034

#### Refinement

*R*[*F* ^2^ > 2σ(*F* ^2^)] = 0.050
*wR*(*F* ^2^) = 0.130
*S* = 1.07
3978 reflections
238 parameters
6 restraints
H-atom parameters constrained
Δρ_max_ = 0.80 e Å^−3^
Δρ_min_ = −0.51 e Å^−3^

### 

Data collection: *SMART* (Bruker, 1998 ▶); cell refinement: *SAINT* (Bruker, 1998 ▶); data reduction: *SAINT*; program(s) used to solve structure: *SHELXS97* (Sheldrick, 2008 ▶); program(s) used to refine structure: *SHELXL97* (Sheldrick, 2008 ▶); molecular graphics: *SHELXTL* (Sheldrick, 2008 ▶); software used to prepare material for publication: *SHELXTL*.

## Supplementary Material

Crystal structure: contains datablocks I, global. DOI: 10.1107/S1600536809048594/kj2135sup1.cif

Structure factors: contains datablocks I. DOI: 10.1107/S1600536809048594/kj2135Isup2.hkl

Additional supplementary materials:  crystallographic information; 3D view; checkCIF report

## Figures and Tables

**Fig. 1.**
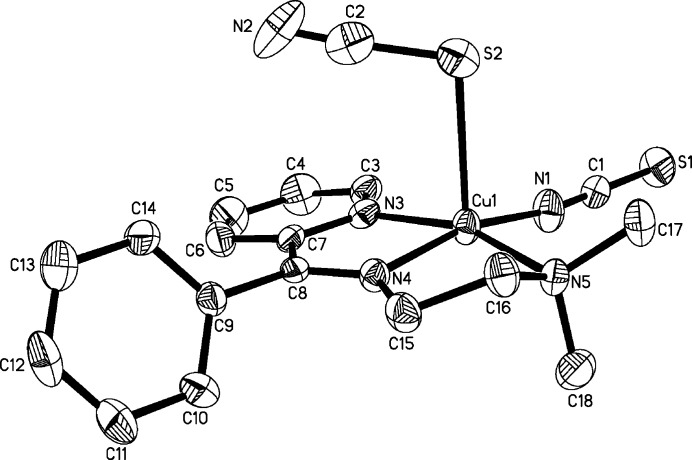
A view of the molecular structure of (I) with the atom-numbering scheme and 30% displacement ellipsoids. All H atoms were omitted for clarity.

## supplementary crystallographic information

### Comment

Schiff base ligands have been extensively studied in coordination chemistry mainly due to their facile synthesis, easily tunable steric, electronic properties and good solubility in common solvents (Shi *et al.*, 2004). Schiff bases have very wide applications as antibacterial, antiviral, antifungal agents (Chandra *et al.*, 2004; Ramesh *et al.*, 2003), and anticancer drugs (Guo *et al.*, 2009). Herein, we report the Cu^II^ complex with the related Schiff base ligand *N*,*N*-dimethyl-N'-(α-(2-pyridyl)benzylidene)ethane-1,2-diamine. The molecular structure of the title compound is shown in Fig. 1. The coordination polyhedron could be determined by using the index τ = (β - α)/60, where β is the largest angle and α is the second. This index is unity for trigonal-bipyramidal geometry and zero for square-pyramidal geometry (Addison *et al.*, 1984). The calculated value for the title compound 0.08, indicating a slightly distorted square pyramidal geometry for the copper atoms. The basal sites are occupied by four nitrogen atoms from one ligand and one NCS^-^ ion with the Cu—N bond lengths ranging from 1.937 (4) to 2.071 (4) Å. In the apical position, another NCS^-^ ion S coordinates to Cu^II^ atom with the Cu—S bond length of 2.679 (2) Å. All bond distances and bond angles have normal values.The dihedral angle between the benzene and pyridine rings is 69.22 (2)°.

### Experimental

2-Benzoylpyridine (0.5 mmol) in 5 ml of methanol solution was added to 5 ml of methanol solution containing 0.5 mmol of *N*,*N*-dimethylethyldiamine. The solution was stirred for 4 h at 60 °C. Then, 0.5 mmol CuSO_4_.5H_2_O in 5 ml distilled water and 1 mmol NH_4_SCN solid was added. The mixture was stirred at 60 °C for 2 h and then cooled and filtered. The filtrate was allowed to slowly evaporate at room temperature. One month later, blue block crystal was obtained.

### Refinement

The structure was refined as a racemic twin with twin ratio 0.56 (3) : 0.44. H atoms on C atoms were positoned geometrically and refined using a riding model (C—H = 0.93 Å for C-Haromatic, C—H = 0.97 Å for *C*-Hmethylene and C—H = 0.96 Å for *C*-Hmethyl). The displacement parameters of atom N2 were mildly restrained to isotropicity (standard uncertainty of 0.01 Å^2^).

### Crystal data

**Table:**

[Cu(NCS)_2_(C_16_H_19_N_3_)]	*D*_x_ = 1.462 Mg m^−^^3^
*M**_r_* = 433.04	Mo *K*α radiation, λ = 0.71073 Å
Orthorhombic, *P*2_1_2_1_2_1_	Cell parameters from 3480 reflections
*a* = 7.6524 (13) Å	θ = 2.7–25.6°
*b* = 9.2048 (15) Å	µ = 1.33 mm^−^^1^
*c* = 27.931 (5) Å	*T* = 294 K
*V* = 1967.4 (6) Å^3^	Block, blue
*Z* = 4	0.30 × 0.24 × 0.20 mm
*F*(000) = 892	

### Data collection

**Table:**

Bruker APEXII CCD area-detector diffractometer	3978 independent reflections
Radiation source: fine-focus sealed tube	3035 reflections with *I* > 2σ(*I*)
graphite	*R*_int_ = 0.034
phi and ω scans	θ_max_ = 26.4°, θ_min_ = 2.3°
Absorption correction: multi-scan (*SADABS*; Sheldrick, 1996)	*h* = −9→9
*T*_min_ = 0.748, *T*_max_ = 1.000	*k* = −11→9
11093 measured reflections	*l* = −34→31

### Refinement

**Table:**

Refinement on *F*^2^	Primary atom site location: structure-invariant direct methods
Least-squares matrix: full	Secondary atom site location: difference Fourier map
*R*[*F*^2^ > 2σ(*F*^2^)] = 0.050	Hydrogen site location: inferred from neighbouring sites
*wR*(*F*^2^) = 0.130	H-atom parameters constrained
*S* = 1.07	*w* = 1/[σ^2^(*F*_o_^2^) + (0.0567*P*)^2^ + 2.0946*P*] where *P* = (*F*_o_^2^ + 2*F*_c_^2^)/3
3978 reflections	(Δ/σ)_max_ = 0.001
238 parameters	Δρ_max_ = 0.80 e Å^−^^3^
6 restraints	Δρ_min_ = −0.51 e Å^−^^3^

### Special details

**Table:**

Geometry. All e.s.d.'s (except the e.s.d. in the dihedral angle between two l.s. planes) are estimated using the full covariance matrix. The cell e.s.d.'s are taken into account individually in the estimation of e.s.d.'s in distances, angles and torsion angles; correlations between e.s.d.'s in cell parameters are only used when they are defined by crystal symmetry. An approximate (isotropic) treatment of cell e.s.d.'s is used for estimating e.s.d.'s involving l.s. planes.
Refinement. Refinement of *F*^2^ against ALL reflections. The weighted *R*-factor *wR* and goodness of fit *S* are based on *F*^2^, conventional *R*-factors *R* are based on *F*, with *F* set to zero for negative *F*^2^. The threshold expression of *F*^2^ > σ(*F*^2^) is used only for calculating *R*-factors(gt) *etc*. and is not relevant to the choice of reflections for refinement. *R*-factors based on *F*^2^ are statistically about twice as large as those based on *F*, and *R*- factors based on ALL data will be even larger.

### Fractional atomic coordinates and isotropic or equivalent isotropic displacement parameters (Å^2^)

**Table:**

	*x*	*y*	*z*	*U*_iso_*/*U*_eq_	
Cu1	0.26036 (8)	0.95205 (6)	0.07382 (2)	0.04339 (19)	
S1	−0.0307 (2)	0.9109 (2)	−0.07293 (6)	0.0670 (5)	
S2	0.0488 (3)	1.1662 (2)	0.10308 (7)	0.0735 (5)	
N1	0.1524 (7)	0.9185 (6)	0.01206 (16)	0.0609 (14)	
N2	−0.0282 (8)	1.0836 (9)	0.1945 (2)	0.098 (2)	
N3	0.1284 (5)	0.7992 (5)	0.11202 (15)	0.0415 (10)	
N4	0.4058 (5)	0.9435 (5)	0.13148 (14)	0.0391 (9)	
N5	0.4643 (6)	1.0786 (5)	0.04853 (14)	0.0429 (10)	
C1	0.0763 (7)	0.9159 (6)	−0.02339 (18)	0.0422 (12)	
C2	0.0070 (9)	1.1144 (8)	0.1584 (3)	0.071 (2)	
C3	−0.0085 (7)	0.7208 (7)	0.0973 (2)	0.0509 (14)	
H3	−0.0606	0.7416	0.0680	0.061*	
C4	−0.0730 (9)	0.6097 (9)	0.1250 (3)	0.078 (2)	
H4	−0.1645	0.5527	0.1137	0.093*	
C5	−0.0016 (9)	0.5823 (8)	0.1698 (3)	0.077 (2)	
H5	−0.0454	0.5080	0.1889	0.092*	
C6	0.1341 (8)	0.6666 (7)	0.1854 (2)	0.0561 (16)	
H6	0.1821	0.6520	0.2156	0.067*	
C7	0.1993 (6)	0.7740 (6)	0.15551 (17)	0.0388 (12)	
C8	0.3525 (7)	0.8643 (6)	0.16633 (16)	0.0376 (11)	
C9	0.4422 (7)	0.8568 (6)	0.21348 (18)	0.0414 (12)	
C10	0.6136 (9)	0.8125 (9)	0.2169 (3)	0.075 (2)	
H10	0.6737	0.7854	0.1894	0.090*	
C11	0.6961 (10)	0.8079 (9)	0.2600 (3)	0.083 (2)	
H11	0.8103	0.7739	0.2618	0.100*	
C12	0.6143 (10)	0.8520 (9)	0.2999 (2)	0.072 (2)	
H12	0.6727	0.8523	0.3291	0.086*	
C13	0.4464 (12)	0.8959 (10)	0.2973 (2)	0.094 (3)	
H13	0.3889	0.9248	0.3250	0.113*	
C14	0.3591 (10)	0.8986 (9)	0.2545 (2)	0.076 (2)	
H14	0.2433	0.9289	0.2534	0.091*	
C15	0.5594 (7)	1.0370 (7)	0.13190 (19)	0.0511 (14)	
H15A	0.6642	0.9799	0.1265	0.061*	
H15B	0.5699	1.0855	0.1626	0.061*	
C16	0.5371 (8)	1.1467 (6)	0.0926 (2)	0.0508 (14)	
H16A	0.4591	1.2232	0.1034	0.061*	
H16B	0.6493	1.1904	0.0853	0.061*	
C17	0.4088 (9)	1.1937 (7)	0.0152 (2)	0.0620 (17)	
H17A	0.5065	1.2552	0.0080	0.093*	
H17B	0.3179	1.2505	0.0298	0.093*	
H17C	0.3656	1.1505	−0.0137	0.093*	
C18	0.5928 (9)	0.9836 (8)	0.0258 (2)	0.0673 (18)	
H18A	0.5454	0.9456	−0.0034	0.101*	
H18B	0.6207	0.9049	0.0470	0.101*	
H18C	0.6969	1.0380	0.0189	0.101*	

### Atomic displacement parameters (Å^2^)

**Table:**

	*U*^11^	*U*^22^	*U*^33^	*U*^12^	*U*^13^	*U*^23^
Cu1	0.0457 (3)	0.0544 (3)	0.0300 (3)	−0.0063 (4)	−0.0057 (3)	0.0011 (3)
S1	0.0712 (10)	0.0874 (12)	0.0424 (8)	−0.0034 (9)	−0.0207 (8)	−0.0084 (9)
S2	0.0689 (11)	0.0779 (12)	0.0737 (12)	0.0142 (10)	−0.0035 (9)	0.0054 (10)
N1	0.071 (3)	0.078 (4)	0.034 (2)	−0.011 (3)	−0.014 (2)	0.000 (3)
N2	0.075 (4)	0.151 (6)	0.067 (4)	−0.047 (4)	0.029 (3)	−0.049 (4)
N3	0.035 (2)	0.050 (3)	0.040 (2)	−0.003 (2)	0.0030 (19)	−0.007 (2)
N4	0.038 (2)	0.047 (2)	0.032 (2)	−0.002 (2)	−0.0039 (17)	0.000 (2)
N5	0.047 (2)	0.047 (3)	0.035 (2)	−0.005 (2)	0.0029 (19)	0.006 (2)
C1	0.046 (3)	0.043 (3)	0.038 (3)	−0.009 (2)	0.000 (2)	−0.005 (2)
C2	0.054 (4)	0.080 (5)	0.079 (5)	−0.010 (3)	−0.009 (4)	−0.020 (4)
C3	0.041 (3)	0.064 (4)	0.048 (3)	−0.003 (3)	0.004 (3)	−0.002 (3)
C4	0.050 (4)	0.088 (5)	0.095 (6)	−0.030 (4)	−0.005 (4)	0.009 (4)
C5	0.054 (4)	0.083 (5)	0.094 (5)	−0.025 (4)	0.011 (4)	0.027 (4)
C6	0.048 (3)	0.070 (4)	0.050 (3)	0.000 (3)	−0.001 (3)	0.018 (3)
C7	0.032 (3)	0.052 (3)	0.032 (3)	0.004 (2)	0.006 (2)	−0.001 (2)
C8	0.041 (3)	0.042 (3)	0.030 (2)	0.015 (2)	0.002 (2)	−0.003 (2)
C9	0.048 (3)	0.043 (3)	0.033 (3)	0.004 (3)	−0.004 (2)	0.001 (2)
C10	0.058 (4)	0.111 (6)	0.056 (4)	0.034 (4)	−0.006 (3)	0.008 (4)
C11	0.064 (5)	0.124 (7)	0.061 (4)	0.016 (4)	−0.023 (3)	0.016 (4)
C12	0.081 (5)	0.085 (5)	0.050 (4)	−0.014 (4)	−0.026 (4)	0.022 (4)
C13	0.107 (7)	0.134 (8)	0.041 (4)	0.031 (6)	−0.009 (4)	−0.007 (4)
C14	0.069 (4)	0.124 (6)	0.035 (3)	0.044 (4)	−0.006 (3)	−0.010 (4)
C15	0.045 (3)	0.063 (4)	0.045 (3)	−0.004 (3)	−0.011 (2)	0.006 (3)
C16	0.059 (3)	0.051 (3)	0.043 (3)	−0.010 (3)	−0.009 (3)	0.003 (3)
C17	0.071 (4)	0.069 (4)	0.046 (3)	−0.010 (3)	−0.002 (3)	0.019 (3)
C18	0.068 (4)	0.067 (4)	0.067 (4)	0.002 (3)	0.019 (3)	0.002 (3)

### Geometric parameters (Å, °)

**Table:**

Cu1—N1	1.937 (4)	C7—C8	1.468 (7)
Cu1—N4	1.959 (4)	C8—C9	1.487 (7)
Cu1—N3	2.034 (4)	C9—C14	1.365 (8)
Cu1—N5	2.071 (4)	C9—C10	1.376 (8)
Cu1—S2	2.679 (2)	C10—C11	1.361 (9)
S1—C1	1.609 (5)	C10—H10	0.9300
S2—C2	1.648 (9)	C11—C12	1.341 (10)
N1—C1	1.149 (7)	C11—H11	0.9300
N2—C2	1.082 (9)	C12—C13	1.349 (11)
N3—C3	1.337 (7)	C12—H12	0.9300
N3—C7	1.351 (6)	C13—C14	1.369 (9)
N4—C8	1.283 (6)	C13—H13	0.9300
N4—C15	1.457 (7)	C14—H14	0.9300
N5—C18	1.461 (7)	C15—C16	1.501 (8)
N5—C17	1.472 (7)	C15—H15A	0.9700
N5—C16	1.490 (7)	C15—H15B	0.9700
C3—C4	1.373 (9)	C16—H16A	0.9700
C3—H3	0.9300	C16—H16B	0.9700
C4—C5	1.389 (10)	C17—H17A	0.9600
C4—H4	0.9300	C17—H17B	0.9600
C5—C6	1.368 (9)	C17—H17C	0.9600
C5—H5	0.9300	C18—H18A	0.9600
C6—C7	1.386 (8)	C18—H18B	0.9600
C6—H6	0.9300	C18—H18C	0.9600
			
N1—Cu1—N4	165.4 (2)	C7—C8—C9	121.7 (4)
N1—Cu1—N3	98.3 (2)	C14—C9—C10	118.0 (6)
N4—Cu1—N3	79.81 (18)	C14—C9—C8	121.0 (5)
N1—Cu1—N5	96.2 (2)	C10—C9—C8	121.0 (5)
N4—Cu1—N5	82.82 (17)	C11—C10—C9	120.8 (7)
N3—Cu1—N5	160.36 (17)	C11—C10—H10	119.6
N1—Cu1—S2	97.53 (18)	C9—C10—H10	119.6
N4—Cu1—S2	97.02 (13)	C12—C11—C10	120.7 (7)
N3—Cu1—S2	92.79 (13)	C12—C11—H11	119.7
N5—Cu1—S2	98.38 (13)	C10—C11—H11	119.7
C2—S2—Cu1	101.0 (3)	C11—C12—C13	119.3 (6)
C1—N1—Cu1	170.7 (5)	C11—C12—H12	120.4
C3—N3—C7	119.9 (5)	C13—C12—H12	120.4
C3—N3—Cu1	127.0 (4)	C12—C13—C14	121.2 (7)
C7—N3—Cu1	113.0 (3)	C12—C13—H13	119.4
C8—N4—C15	125.9 (4)	C14—C13—H13	119.4
C8—N4—Cu1	117.8 (3)	C9—C14—C13	120.0 (7)
C15—N4—Cu1	116.2 (3)	C9—C14—H14	120.0
C18—N5—C17	110.5 (5)	C13—C14—H14	120.0
C18—N5—C16	111.0 (5)	N4—C15—C16	107.4 (4)
C17—N5—C16	109.1 (4)	N4—C15—H15A	110.2
C18—N5—Cu1	108.6 (4)	C16—C15—H15A	110.2
C17—N5—Cu1	113.7 (4)	N4—C15—H15B	110.2
C16—N5—Cu1	103.7 (3)	C16—C15—H15B	110.2
N1—C1—S1	179.5 (6)	H15A—C15—H15B	108.5
N2—C2—S2	176.5 (8)	N5—C16—C15	111.3 (5)
N3—C3—C4	120.7 (6)	N5—C16—H16A	109.4
N3—C3—H3	119.6	C15—C16—H16A	109.4
C4—C3—H3	119.6	N5—C16—H16B	109.4
C3—C4—C5	120.0 (6)	C15—C16—H16B	109.4
C3—C4—H4	120.0	H16A—C16—H16B	108.0
C5—C4—H4	120.0	N5—C17—H17A	109.5
C6—C5—C4	118.9 (6)	N5—C17—H17B	109.5
C6—C5—H5	120.6	H17A—C17—H17B	109.5
C4—C5—H5	120.6	N5—C17—H17C	109.5
C5—C6—C7	119.1 (6)	H17A—C17—H17C	109.5
C5—C6—H6	120.5	H17B—C17—H17C	109.5
C7—C6—H6	120.5	N5—C18—H18A	109.5
N3—C7—C6	121.3 (5)	N5—C18—H18B	109.5
N3—C7—C8	114.1 (4)	H18A—C18—H18B	109.5
C6—C7—C8	124.5 (5)	N5—C18—H18C	109.5
N4—C8—C7	114.8 (4)	H18A—C18—H18C	109.5
N4—C8—C9	123.5 (5)	H18B—C18—H18C	109.5
			
N1—Cu1—S2—C2	134.4 (3)	C3—C4—C5—C6	−0.7 (11)
N4—Cu1—S2—C2	−44.5 (3)	C4—C5—C6—C7	−1.7 (10)
N3—Cu1—S2—C2	35.6 (3)	C3—N3—C7—C6	0.7 (8)
N5—Cu1—S2—C2	−128.2 (3)	Cu1—N3—C7—C6	−175.7 (4)
N1—Cu1—N3—C3	−8.9 (5)	C3—N3—C7—C8	177.9 (4)
N4—Cu1—N3—C3	−174.2 (5)	Cu1—N3—C7—C8	1.5 (5)
N5—Cu1—N3—C3	−146.0 (5)	C5—C6—C7—N3	1.7 (9)
S2—Cu1—N3—C3	89.2 (4)	C5—C6—C7—C8	−175.2 (5)
N1—Cu1—N3—C7	167.2 (4)	C15—N4—C8—C7	−176.7 (5)
N4—Cu1—N3—C7	1.9 (3)	Cu1—N4—C8—C7	8.0 (6)
N5—Cu1—N3—C7	30.1 (7)	C15—N4—C8—C9	0.2 (8)
S2—Cu1—N3—C7	−94.7 (3)	Cu1—N4—C8—C9	−175.1 (4)
N1—Cu1—N4—C8	−89.6 (9)	N3—C7—C8—N4	−6.1 (6)
N3—Cu1—N4—C8	−5.7 (4)	C6—C7—C8—N4	171.0 (5)
N5—Cu1—N4—C8	−176.5 (4)	N3—C7—C8—C9	176.9 (4)
S2—Cu1—N4—C8	85.9 (4)	C6—C7—C8—C9	−6.0 (8)
N1—Cu1—N4—C15	94.7 (9)	N4—C8—C9—C14	118.4 (7)
N3—Cu1—N4—C15	178.6 (4)	C7—C8—C9—C14	−64.9 (7)
N5—Cu1—N4—C15	7.8 (4)	N4—C8—C9—C10	−59.2 (8)
S2—Cu1—N4—C15	−89.8 (4)	C7—C8—C9—C10	117.5 (7)
N1—Cu1—N5—C18	−75.9 (4)	C14—C9—C10—C11	1.3 (11)
N4—Cu1—N5—C18	89.4 (4)	C8—C9—C10—C11	179.0 (7)
N3—Cu1—N5—C18	61.5 (7)	C9—C10—C11—C12	−2.7 (13)
S2—Cu1—N5—C18	−174.5 (4)	C10—C11—C12—C13	2.5 (13)
N1—Cu1—N5—C17	47.6 (4)	C11—C12—C13—C14	−1.1 (14)
N4—Cu1—N5—C17	−147.1 (4)	C10—C9—C14—C13	0.1 (11)
N3—Cu1—N5—C17	−175.0 (5)	C8—C9—C14—C13	−177.6 (7)
S2—Cu1—N5—C17	−51.0 (4)	C12—C13—C14—C9	−0.2 (14)
N1—Cu1—N5—C16	165.9 (4)	C8—N4—C15—C16	−159.8 (5)
N4—Cu1—N5—C16	−28.7 (3)	Cu1—N4—C15—C16	15.6 (6)
N3—Cu1—N5—C16	−56.7 (6)	C18—N5—C16—C15	−70.3 (6)
S2—Cu1—N5—C16	67.3 (3)	C17—N5—C16—C15	167.6 (5)
C7—N3—C3—C4	−3.2 (8)	Cu1—N5—C16—C15	46.1 (5)
Cu1—N3—C3—C4	172.7 (5)	N4—C15—C16—N5	−41.5 (6)
N3—C3—C4—C5	3.2 (10)		
